# Scenario construction and evolutionary analysis of nonconventional public health emergencies based on Bayesian networks

**DOI:** 10.3389/fpubh.2025.1489904

**Published:** 2025-02-07

**Authors:** Yutao Zhu, Qing Yang, Lingmei Fu, Chun Cai, Jinmei Wang, Ling He

**Affiliations:** ^1^School of Management, Wuhan University of Technology, Wuhan, China; ^2^School of Safety Science and Emergency Management, Wuhan University of Technology, Wuhan, China; ^3^College of Emergency Management, Nanjing Tech University, Nanjing, China; ^4^School of Automotive Engineering, Wuhan University of Technology, Wuhan, China

**Keywords:** unconventional public health emergencies, scenario evolution, Bayesian networks, emergency response, COVID-19

## Abstract

**Objectives:**

The objective was to aggregate the various scenarios that occur during nonconventional public health emergencies (NCPHEs) and analyze the evolutionary patterns of NCPHEs to better avoid risks and reduce social impacts. The aim was to enhance strategies for handling NCPHEs.

**Study design:**

News reports were crawled to obtain the scenario elements of NCPHEs and categorized into the spreading stage or derivation stage. Finally, the key scenario nodes and scenario evolution process were analyzed in combination with a corresponding emergency response assessment of each scenario by experts.

**Methods:**

Dempster–Shafer (DS) theory and Bayesian networks (BNs) were applied for data reasoning, and a spread-derived coupled scenario–response theoretical model of NCPHEs for major public health emergencies was constructed. The scenario evolution path of COVID-19 was derived by combining seven types of major scenario states and corresponding emergency response measures extracted from 952 spreading scenarios.

**Results:**

The 26 NCPHE spread scenarios and 41 NCPHE derivation scenarios were summarized. Optimized and pessimistic NCPHE scenario pathways were generated by combining the seven major spreading scenarios to help decision makers predict the development of NCPHEs and take timely and effective emergency response measures for key scenario nodes.

**Conclusion:**

This study provides a new approach for understanding and managing NCPHEs, emphasizing the need to consider the specificity and complexity of such emergencies when developing decision-making strategies. Our contextual derivation model and emergency decision-making system provide practical tools with which to enhance NCPHE response capabilities and promote public health and safety.

## 1 Introduction

The COVID-19 outbreak was a major nonconventional public health emergency (NCPHE) with unprecedented spread, extensive infections, and significant challenges in prevention and control. The outbreak tested healthcare institutions, citizens, governments, and enterprises, as well as governance mechanisms at all levels in affected countries (1). This led to extreme tests and destructive experiments being carried out on various subjects, such as healthcare institutions, people, governments, and enterprises, as well as the governance mechanisms and institutions at all levels in countries in the middle of the outbreak (2, 3).

COVID-19 was an NCPHE that was characterized by suddenness, complexity, uncertainty, and broad public impact, alongside significant secondary derivative hazards that were both vast and destructive. Given the novel nature of the virus, its potential for mutation, and its high morbidity and mortality rates, Hao X and other scholars have studied COVID-19. Notably, they identified two distinct features on the basis of 32,583 confirmed cases, namely, a high level of insidiousness and a high transmission rate (4). Thus, a dynamic policy response is needed to effectively mitigate risks.

Recent NCPHE crises, including influenza A (H1N1), Zika, MERS, and Ebola, have been frequent, unpredictable, and destructive, revealing significant information gaps and data availability issues (5). These patterns have intensified concerns about future NCPHEs, highlighting the urgent need for strong policy measures to mitigate risks.

Three years after the onset of the COVID-19 epidemic, most global economies are still influenced by the adverse effects of urban lockdowns and economic disruptions (6). To effectively manage NCPHEs such as COVID-19, scenario forecasting can be used as a more dynamic approach than traditional methods. In this approach, outcomes are predicted under specific assumptions, incorporating key scenario factors that allow for a timely response to potential situations arising during a health crisis (7, 8). Uncertainties such as behavioral shifts, new government measures, and natural disasters are considered in scenario forecasting, thus enhancing our ability to plan and implement effective emergency responses.

In response to COVID-19, countries have adopted nonpharmacological interventions (NPISs), such as community isolation, social distancing, controlling the movement of people, closing schools, rapid virus testing, close contact tracing, and the establishment of square-cabin hospitals (9, 10). These interventions can be divided into two categories, namely, blocking and mitigating modes, which can also be described as rigid and flexible modes, respectively (11). The blocking model (rigid model) requires higher costs in the short term due to the enforcement of interventions to avoid significant long-term health and economic losses. On the other hand, proponents of the mitigation model (flexible model) argue that the blocking model cannot fully eradicate COVID-19. In later stages, when the epidemic subsides, high-intensity interventions may lead to overdefense, adversely impacting both population welfare and economic development and ultimately reducing the effectiveness of subsequent interventions (12, 13).

Scenario forecasting can be used to analyze different dimensions of NCPHE-specific past and future scenarios simultaneously given the relevant NPISs. Scenario forecasting aids in controlling emergency response for NPISs during NCPHEs and can support long-term decision-making better than traditional forecasting methods can (14).

Therefore, the effective prediction and projection of processes in various NCPHE scenarios (spread, derivation, and coupling) and the generation of contingency plans can not only facilitate communication between key public health and emergency agencies but also aid in identifying critical issues and effective responses. This approach also supports the global restructuring of governance systems for unconventional public health emergencies.

The “scenario–response” approach has become a popular approach in emergency response research, especially with respect to responding to NCPHEs, which are difficult to predict and are characterized by a high degree of complexity or low probability of occurrence. The “scenario–response” approach was first used in the preparation of China’s emergency response plan; the idea is to use scenarios to express routine emergencies as the initial goal, the prediction of future scenarios as the intermediate goal, and the proposal of response options under specific scenarios as the final goal. The “scenario–response” method also helps government departments carry out prevention and emergency preparedness work for different scenarios [social security (15), education (16), management (17), health (18), climate adaptation (19), ecosystems (20), and public health (21)] under the premise of risk prevention and early warning provision, with the aim of carrying out prevention and emergency preparedness work.

Many scholars have conducted exploratory research at the theoretical level and applied research at the practical level in tasks such as problem definition, elemental composition, and expression to evaluate the evolution mode of scenarios. In terms of the theoretical level, Kahn and Anthony defined a scenario as a collection of hypothetical future events constructed to elucidate possible causal chains of events and their decision points (22). On this basis, Durance and Godet argued that one needs to satisfy the requirements of relevance, coherence, likelihood, importance, and transparency to label a scenario (23). On the basis of the classification of scenarios into predictive, exploratory and normative categories, Börjeson et al. introduced the concepts of external scenarios and internal scenarios (24).

The commonly used scenario analysis methods include the scenario axis technique, with which it is possible to establish scenarios in which trends and key uncertainties for past events are transformed into a variety of plausible trends to help describe different possible future states of the world (25). Another method is the 2 × 2 scenario matrix method, in which a matrix is derived from a selection of two causally independent key drivers ranging from extremely positive (favorable) developments to extremely negative outlooks (26, 27). Morphological analysis spans the entire field of possibilities, and relevant scenarios are constructed. Notably, Johansen used morphological analysis to structure scenarios (28). Expert analysis (such as with Delphi or Renner Abacus) is used to assign probabilities and reduce uncertainty, and multicriteria analysis is used to identify and evaluate strategic options.

In summary, scenario representation for nonconventional emergencies can be summarized as the collection of current scenario element status information and the sum of development trends. The key to the emergency management of NCPHEs is the rationality of response decisions. Therefore, with respect to how to correctly represent the status of various scenarios, whether the composition of scenario elements is reasonable, and whether the assessment approach is appropriate, each scenario deduction link is particularly important.

In terms of applied research at the practical level, the U.S. COVID-19 Scenario Modeling Hub (SMH) uses scenario planning models to simulate changes in uncertainty in the epidemiology of COVID-19 and its response measures, combined with future changes under specific conditions (such as intervention policies), to provide long-term guidance (29). Oteros-Rozas et al. used participatory scenario planning (PSP) to study the socioecological environment. Robin B et al. proposed the use of participatory prospective analysis (PPA) to construct scenarios to increase the capacity of local communities and organizations. Srivastava et al. used three machine learning methods, namely, linear regression (LR), sequential minimal optimization (SMO) regression, and the M5P algorithm, to predict COVID-19 scenarios (30). Similarly, scenario simulations have been applied to assess natural disaster events under extreme climate conditions, such as rainstorms and floods, and during emergencies, such as those related to water pollution, oil depot explosions and fires, and food safety incidents (31, 32).

Most studies of NCPHE scenario simulations have focused on single-scenario analysis, used quantitative models to predict the short-term trends of public health events, or analyzed scenario factors from a risk perspective (33–35). However, these methods seldom integrate relevant countermeasures or decision-making strategies.

On the basis of scenario evolution theory and the Bayesian network (BN) model, a scenario deduction model for NCPHEs was constructed. It can be used to effectively address new “what-if” problems that may arise during public health outbreaks due to behavioral changes or the implementation of new intervention measures. Compared with traditional prediction models, the scenario evolution model we constructed on the basis of a BN incorporates multiple scenario assumptions that support reasoning in cases with incomplete, imprecise, or uncertain information and can therefore meet long-term decision-making needs.

## 2 Methodology

A BN is a model for uncertainty knowledge representation and reasoning that is based on probabilistic analysis and graph theory; it is currently one of the most effective models used in the field of uncertainty knowledge representation and reasoning. BNs consist of nodes (variables) and directed edges (conditional dependencies). Each node represents a random variable, and directed edges between nodes represent conditional dependencies (pointing from parent to child), expressing the strength of the relationship in terms of conditional probabilities. Bayesian networks perform inference by utilizing Bayes’ theorem and quantify these relationships through probability distributions.

In a BN model, if the set of parent nodes of a particular node is established and given the conditional independence assumption inherent to BNs, then the node in question is statistically independent of all nondescendant nodes. Under these conditions, the joint probability distribution can be formulated as follows:


(1) $$\begin{array}{l} P\left(x_{1},x_{2},\ldots ,x_{n}\right)=P\left(x_{1}\right)P\left(\;x_{2}|x_{1}\right)P\left(\;x_{3}|x_{1},x_{2}\right)\cdots \\ P\left(\;x_{n}|x_{1},x_{2},\cdots x_{n-1}\right)=\prod_{i=1}^{n}P\left(x_{1}|P\left(\prod x_{n}\right)\right) \end{array}$$

To overcome the limitations of expert knowledge and the influence of personal preferences on the accuracy of empirical probability, Dempster–Shafer (DS) theory (36) is used to integrate the empirical probability values and uncertain information given by experts to reduce their subjective effects and ensure the reliability and rationality of node probabilities.

Based on an improved DS theory, the empirical probability values given by various experts are quantitatively calculated, which can greatly reduce the subjective effect and improve the accuracy of empirical probability. The improved fusion formula is as follows (37):


(2) $$m_{DS}\left(A\right)=\{\begin{array}{c} \frac{\sum_{A_{i}\cap B_{j}\cdots =A}m_{1}\left(A_{i}\right)m_{2}\left(B_{i}\right)}{1-K}+f\left(A\right)\;A\neq \emptyset \\ \;0\;\;A=\emptyset \end{array}$$

f(A) = k·q(A) in the formula is the probability distribution function of evidence conflict; that is, the conflict degree K between each piece of evidence is distributed to each element in Matrix A. Therefore, the probability distribution formula satisfies $\sum_{A\subset \theta }f\left(A\right)=K$, where $q\left(A\right)=\frac{\sum_{i=1}^{n}m_{i}\left(A\right)}{n}$ allocates K to A according to this ratio.

For scenario variable with parent nodes, its occurrence probability is determined according to the expert’s scoring (named conditional probability), that is, the probability values of this scenario variable under the occurrence and nonoccurrence of its parent scenario node. P(S_i_) represents the parent node set of the situation state node Si, and the corresponding formula can be expressed as follows:


(3) $$P\left(S_{1},S_{2},\cdots ,S_{n}\right)=\prod_{i=1}^{n}P\left(S_{i}|P_{a}\left(S_{i}\right)\right)$$

In DS theory, upper and lower boundary probabilities are used to solve the multivalued mapping problem and fuse multiple data sources or expert judgments to reduce the bias of a single source, thus solving problems related to the characterization and fusion of uncertain information. By using DS theory in conjunction with BNs, we are not only able to attenuate uncertainty in the absence of precise probabilistic information but also enhance the robustness of BN inference. Notably, in scenarios with conflicting evidence from multiple sources, we can effectively avoid inference errors due to the inconsistency of information. By combining these two approaches, our scenario evolution model for NCPHEs can maintain high inference accuracy and provide reliable support for decision making in cases with uncertainty or insufficient data.

## 3 Data collection

### 3.1 NCPHE scenario construction and evolutionary element data collection

For NCPHEs, the International Health Regulations (IHR 2005), which were revised in 2005 by the World Health Organization (WHO), define an international public health emergency as follows: “A public health emergency of international concern is an unusual event that poses a public health risk to other countries through the international spread of disease and may require a coordinated international response” (38). The U.S. Centers for Disease Control and Prevention (CDC) define an NCPHE as any event that may have a significant impact on the health of a population, such as natural disasters, disease outbreaks, and bioterrorist attacks (39). This definition emphasizes the potential impact of the event on public health and is not specific to an international context. Other countries, such as China, define an NCPHE as a health threat that exceeds the ability of conventional public health and medical resources to respond to it; such a public health event typically includes major infectious disease outbreaks, mass unexplained illnesses, major food and occupational poisonings, and other situations that seriously affect public health and cause serious damage to the public health of the community.

**Figure 1:**
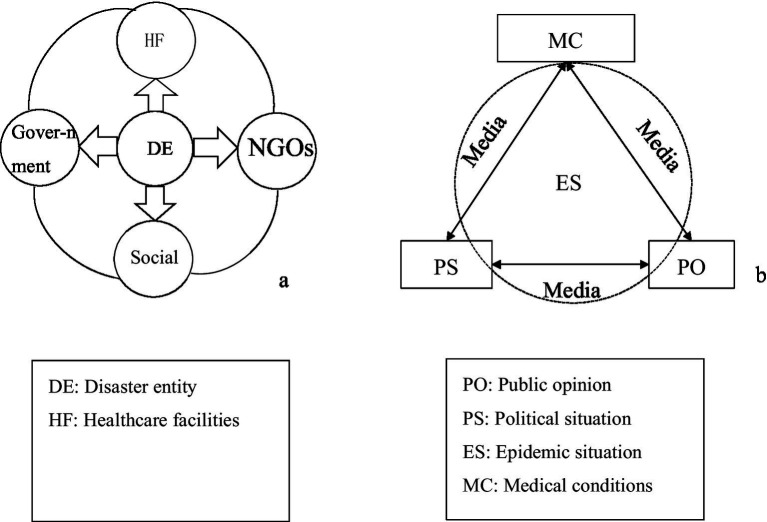
Framework diagram of the relationships among the elements of an unconventional public health emergency scenario.

The scenario elements in the emergency prevention and control process for NCPHEs can be systematically delineated from three perspectives (illustrated using a major epidemic as a case study), namely, disaster-affected entities [such as the government, societal elements such as residents and businesses, and nongovernmental organizations (NGOs)], disaster agents (such as viruses and epidemic-related materials), and disaster-response entities (medical and health institutions, alongside public service organizations) (40). Figure 1A illustrates the interrelationships among these three categories of actors. Through media (the internet), the three types of entities engage in energy and information exchange. Figure 1B is a schematic diagram illustrating the interactions of information and energy among these entities. This scenario element description is used to summarize the various scenarios that can arise during the evolution of NCPHEs.

Second, COVID-19 served as the empirical research focus for collecting and analyzing data for typical NCPHE scenarios. The epidemic was characterized by an extensive impact, a high degree of concealment, a rapid transmission rate, and the involvement of multiple stakeholders. Traditional emergency planning procedures are inadequate for the timely and effective management of such events. Therefore, the COVID-19 outbreak exemplifies a typical unconventional public health emergency, providing a pertinent case study with which to validate the accuracy and efficacy of the model proposed in this study.

**Figure 2:**
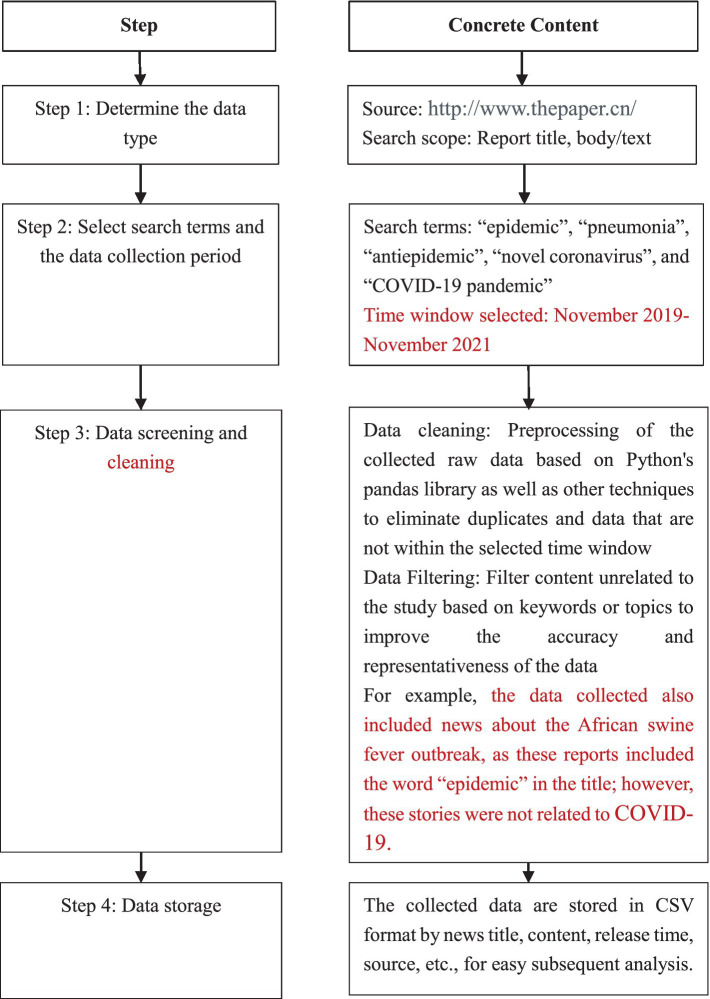
NCPHE data collection flowchart.

ThePaper is a well-known news medium in China, specializing in in-depth news reporting and public affairs, with content of high authority and credibility. The news content it publishes usually undergoes strict editing and review procedures to ensure the accuracy and authenticity of the information. Tencent News and Baidu News are websites that are more oriented toward news aggregations, providing information mainly by reproducing content from other news sources. The original reports of ThePaper, especially with respect to NCPHEs, can provide a detailed and analytical perspective for scenario analysis and modeling. Therefore, ThePaper was chosen as the news collection source. The flow of the data collection steps is shown in Figure 2.

Following the steps above, using the Houyi collector tool, a total of 9,132 records were initially retrieved. The dataset was refined by excluding news events prior to 2019, events unrelated to the epidemic scenarios and duplicate reports. After screening, 1,504 relevant cases of COVID-19-related news reports were identified for analysis.

Finally, drawing from disaster system theory, a disaster situation was determined to comprise three elements, namely, the disaster-causing factor, the disaster carrier, and the disaster-conceiving environment, which can be formulated as situation = {hazard, carrier, environment} (41). On the basis of whether they are directly or indirectly affected by epidemics, the identified scenarios were categorized into 26 types of spreading scenarios and 41 types of derived scenarios (37 types of negative scenarios and 4 types of positive scenarios). After thorough comparative analyses, 952 spreading scenarios and 552 derived scenarios were identified. The basic descriptions of these scenarios are presented in Appendix A.

**Figure 3:**
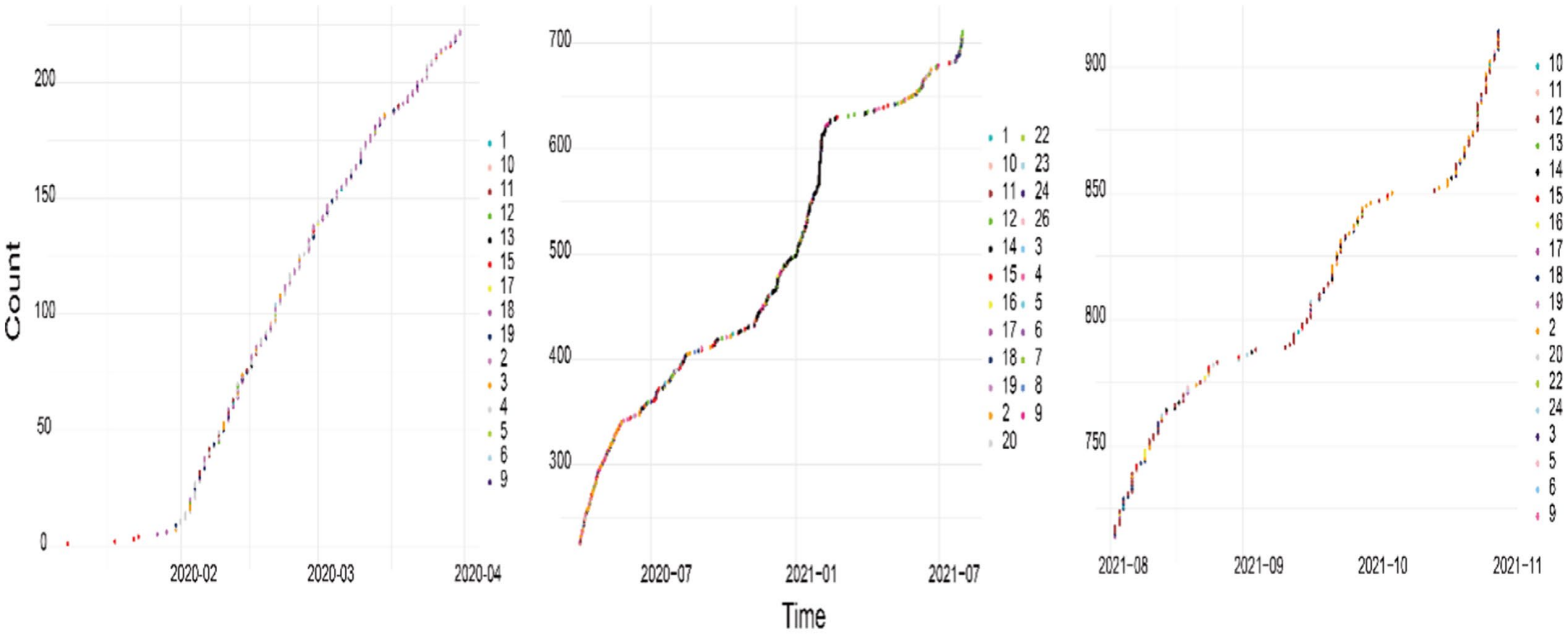
Graph of the number of different scenarios in each period.

From the beginning of the epidemic to the resumption of regular socioeconomic activities, China’s epidemic prevention and control included three phases: the outbreak blocking phase, the normalized prevention and control exploratory phase, and the “dynamic zero” phase of whole-chain precision prevention and control. The time nodes of these three phases are defined as April 2020, August 2021, and November 2021, respectively; thus, the scenarios were divided into periods according to these three time nodes. The spreading scenarios and the number of occurrences in these three periods are shown in Figure 3.

Figure 3 clearly shows that in the initial phase, news reports covered primarily influenza or other viruses, which was attributed to the similarity of the symptoms of these viruses to those of COVID-19. This was followed by increased coverage of the surge in infection rates (S2) and the scarcity of medical supplies and personnel (S3 and S4). In the subsequent period, the focus shifted due to the government’s adaptive emergency responses, which moved away from stringent quarantine measures. During this time, the growth rate of S2 reports noticeably slowed, whereas reports on superspreader events caused by virus carriers (S12) and risks associated with virus transmission through food (particularly cold chain transport) (S14) increased.

In the final phase, the narrative continued to be dominated by reports on S2 and S12. However, there was a notable shift in reporting trends; the frequency of reports regarding individual-induced virus transmission (S12) surpassed that of reports regarding the surge in infection rates (S2). Attention also increasingly turned toward incidents involving inappropriate statements and rumors (S18).

### 3.2 Bayesian scenario network construction for NCPHEs

**Figure 4:**
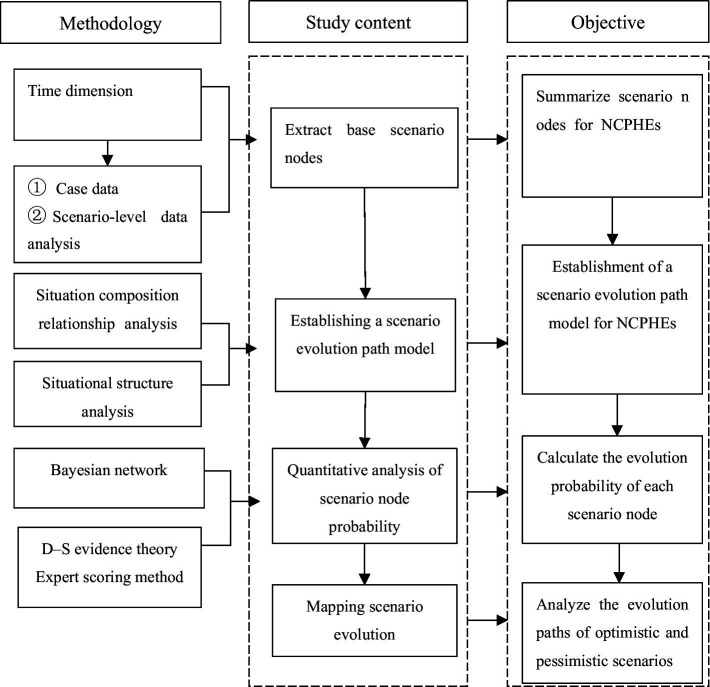
Flowchart for the construction of a scenario network for NCPHEs.

The Bayesian scenario network construction process for NCPHEs can be divided into three steps in general; the specific flow chart is shown in Figure 4.

1. Determination of network key node variables. By analyzing the historical cases of NCPHEs and expert knowledge to determine the key nodes of an event, the results of the quantitative analysis can be used to establish network node variables. On the basis of the scenario analysis, the key element nodes were counted, and then the domain expert knowledge was integrated to score and determine the type and value range of the node variables.

The types of node variables in the model were classified into scenario state nodes at a certain moment and scenario disappearance state nodes, where the scenario state nodes indicate the results of scenario state evolution at a certain moment and the scenario disappearance nodes indicate the disappearance of the crisis state, which suggests that the event is developing in a positive direction.

2. Determination of the relationships among network nodes. To determine the scenario node variables, the relationships among node variables were determined according to the historical timing of an event and the logical relationships among the node variables; additionally, directed edges were used to connect the causally related node variables, constituting an interrelated network model of the scenario states.

The scenario evolution of an NCPHE is essentially a successive unfolding of the BN on the basis of a certain timeline; therefore, if the entire NCPHE evolution process is divided into a collection of n scenarios at time T, then S1 represents the initial scenario in the first critical phase, Si represents the scenario state at time Ti, Di (the disposal target) represents the disposal target at time Ti, and Mi denotes the contingency measure (emergency measure, Mi). The variable types and value sets of each scenario node are shown in Table 1.

**Table 1:** Variable types and sets of values for key scenario nodes.

Node variable name	Node variable type	The set of node values
Scenario state (S)	Boolean variable	{True (T), False (F)}
Emergency measure (M)	Binary ordinal variable	{Good (G), Bad (B)}
Disposal objective (D)	Binary ordinal variable	{Positive (P), Negative (N)}

**Figure 5:**
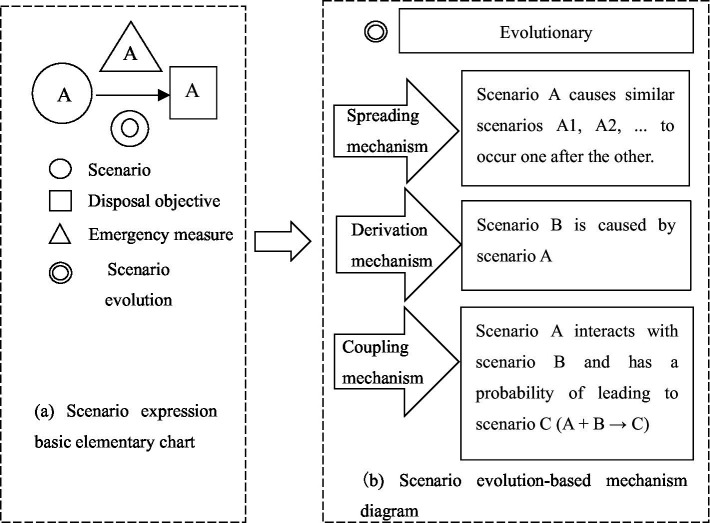
NCPHE scenario evolution and network expression map.

**Figure 6:**
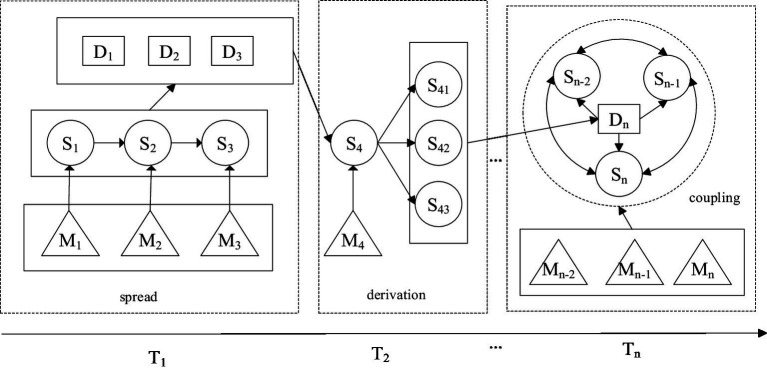
Scenario evolution pathway for NCPHEs.

On the basis of the scenario modeling of NCPHEs (42), as shown in Figure 5, an SDM model is constructed with these three key nodes for the three evolution modes of spreading, derivation, and coupling to describe the BN evolution process of NCPHEs scenarios, as shown in Figure 6.

In Figure 5, the fundamental elements used to represent scenarios in emergency management. The figure shows the mechanisms by which scenarios evolve, including the spreading, derivation and coupling Mechanism.

Figure 6 illustrates how scenarios evolve and interact through various mechanisms, emphasizing the dynamic and interconnected nature of scenarios in the context of emergency management.

**Figure 7:**
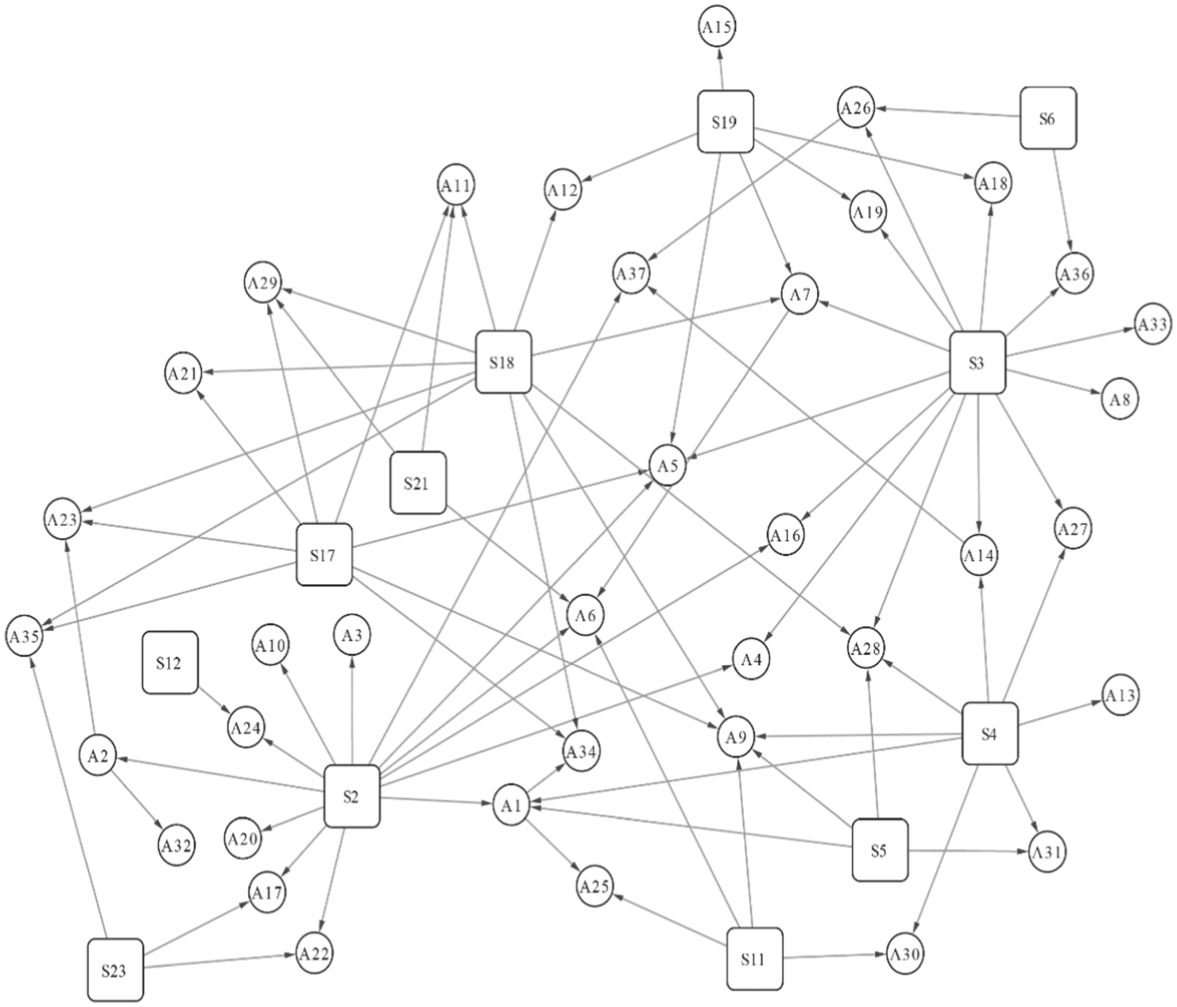
Derivation of NCPHE scenarios.

By combining the information in Figure 6 and Appendix A, the derivation diagram of NCPHE scenarios can be drawn via Cytoscape, as shown in Figure 7, in which a spreading scenario of NCPHEs is shown in the box and the derived scenarios labeled on the basis of the spreading scenario results are shown in the circle. In this study, we focus on the scenario evolution process during the spreading process of NCPHEs.

3. Determination of the probabilities of network nodes. To analyze the evolution of scenario states and calculate the occurrence probability of network node variables in each scenario, it is necessary to first determine the conditional probability of node variables with parent nodes and the prior probability of node variables without parent nodes in the network on the basis of expert knowledge.

## 4 Analysis of results

### 4.1 Analysis of the evolution path of major epidemic scenarios

Through the analysis of historical cases and data collection, combined with the top events reported in the news shown in Figure 3 and the derivative diagram of NCPHEs in Figure 6, six key scenario nodes, namely, S2, S3, S4, S12, S14, and S18, were extracted. However, from the analysis of the scenario elements and nodes, the affected objects and disaster relief agencies in scenarios S2, S3, S4, and S12 were all consistent and directly affected by certain objects, and the affected objects were all associated with the public and society. The disaster relief agencies were mainly medical and health institutions. The affected bodies in scenarios S14 and S18 also included enterprises, which were indirectly affected by disaster bodies. Therefore, S14 and S18 were not considered in the scenario evolution path in the present study. The affected bodies and disaster relief agencies in S5, S6, and S7 were consistent with those in scenarios S2, S3, S4, and S12. Therefore, S2, S3, S4, S5, S6, S7, and S12 were set as node variables in the major epidemic scenario network. Combined with the development and evolution of the epidemic crisis at different stages, the evolution path of this major epidemic encompassed 7 scenario states, 7 emergency measures, and 7 disposal targets (see Table 2).

**Table 2:** COVID-19 scenario elements.

Scenario state (S)	Disposal target (D)	Emergency measure (M)
S_2_: The number of people infected with severe local epidemics has increased sharply	D_2_: Nucleic acid testing, centralized isolation and treatment of virus-infected people	M_2_: Temporarily close businesses, schools, shopping malls and other places with high exposure risk (44)
S_3_: Shortage of medical supplies and protective equipment	D_3_: Provinces dispatch masks and replenish medical supplies	M_3_: Increasing the production of medical supplies and strengthening their upstream and downstream chains
S_3end_: Crisis disappeared 3		
S_4_: Medical personnel infected with the virus	D_4_: Timely isolation of infected medical personnel	M_4_: Enhancement of protective measures and training for medical personnel, while ensuring that they have adequate rest periods
S_4end_: Crisis disappeared 4		
S_5_: Insufficient medical personnel	D_5_: Dispatch of medical personnel from other areas for medical team support	M_5_: Remote use of the internet by off-site healthcare workers
S_6_: Insufficient hospital beds	D_6_: Increase the number of hospital beds	M_6_: Makeshift hospitals (MSHs) erected in Fangcang, Huoshenshan, and Leishenshan
S_6end_: Crisis disappeared 6		
S_7_: Misdiagnosis of virus carriers	D_7_: Improved diagnostic methods	M_7_: Combined use of multiple diagnostic tools
S_7end_: Crisis disappeared 7		
S_12_: The emergence of superspreaders	D_12_: Controlling the movement of people	M_12_: Home isolation measures
S_12end_: Crisis disappeared 12		

**Figure 8:**
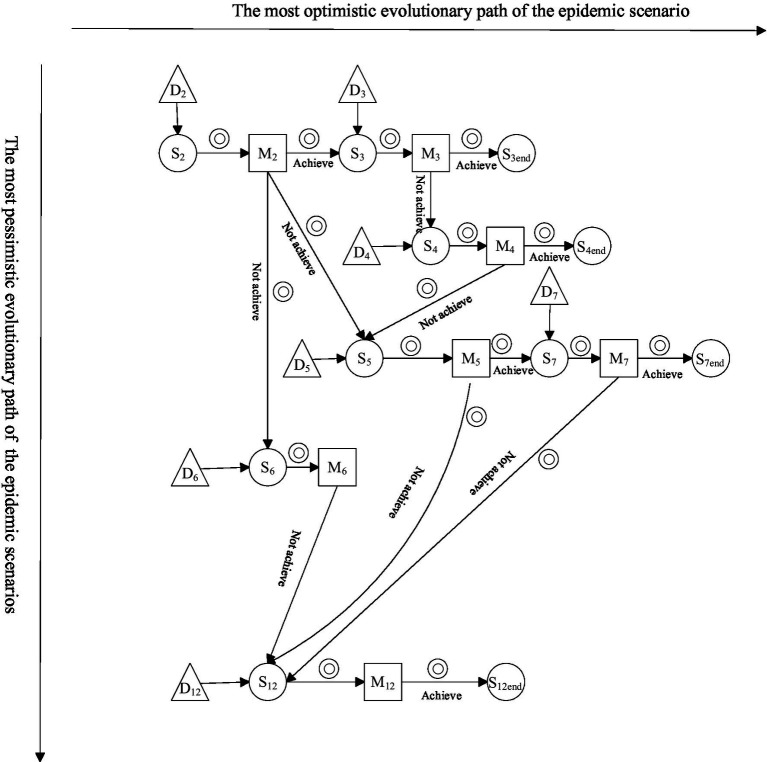
Schematic diagram of the evolutionary path of the major outbreak scenario in 2020.

Owing to the emergence of a localized surge in the number of infected people in S2, the whole scenario chain evolved over time, while the corresponding emergency measures (M2, M3, M4, M5, M6, M7, and M12) were implemented given the regulation and intervention effects linked to the different disposal objectives (D2, D3, D4, D5, D6, D7, and D12), with the goal of scenarios evolution in a specific direction toward positive outcomes, reflecting the evolution path. The path in this case is schematically shown in Figure 8.

As shown in Figure 8, during the development of public health emergencies, the evolutionary path of scenarios changes due to the different implementation effects of emergency response measures. For example, if emergency measure D3 is implemented in scenario status S3, and if D3 is effective, then scenario S3 will develop toward the expected goal; i.e., the crisis may end, and the scenario may disappear. However, if the implementation of D3 is ineffective, then S3 may evolve into a new scenario, such as S4. For scenario S4, emergency measure M4 is implemented, and if the results of M4 are favorable, then the scenario ends.

In Figure 8, there are 11 scenario states, in which S3end, S4end, S7end, and S12end, which have no parent nodes, are crisis disappearance scenarios, and the other nodes are scenario states. In this scenario evolution network, the horizontal arrows indicate the optimal evolution path achieved under the joint effect of emergency measures and treatment goals, such as S2 → S3 → S3end; i.e., the surge in the number of infected people disappears in the case of the effective implementation of measures D2 (complete isolation and admission of virus-infected people) and D3 (active replenishment of medical materials, with no gap in medical material availability). The vertical arrows indicate the nonoptimal evolutionary paths, in which emergency measures are not effective and treatment objectives are not fully achieved, such as S3 → S4 → S5 → S12; e.g., when medical supply shortages occur and when medical staff are infected with the virus, insufficient medical staff issues and the potential for superspreader scenarios will exist.

### 4.2 Probability of a major outbreak scenario

In major epidemic Bayesian networks, the estimation of node probabilities is usually challenging because of limited access to data. Therefore, professionals need to assess the probability of each node variable on the basis of their experience and expertise.

Seven domain experts in public health and emergency management were invited to score each node variable, and a Gaussian affiliation function was used to describe the degree of ambiguity of each factor. The central values of occurrence and nonoccurrence of the affiliation function were set to 0.75 and 0.25, respectively (43):


(4) $$y=e^{-\frac{{\left(x-\mu \right)}^{2}}{\sigma^{2}}}$$

where *x* represents the score of each expert for each indicator, μ represents the central value of the function, and σ represents the uncertainty or estimation error of the expert’s score. The smaller σ is, the more reliable the expert’s score is.

This Gaussian affiliation function was used to determine the degree of affiliation of each expert with each indicator on the basis of the occurrence and nonoccurrence ratings, where the value of the affiliation function is the value in the matrix of Formula (4). The probabilities assigned to the seven experts were then fused with Formulas (4), (5), and (6) in the Appendix B. The probability for each node variable was calculated via Dempster’s evidence theory formula.

**Figure 9:**
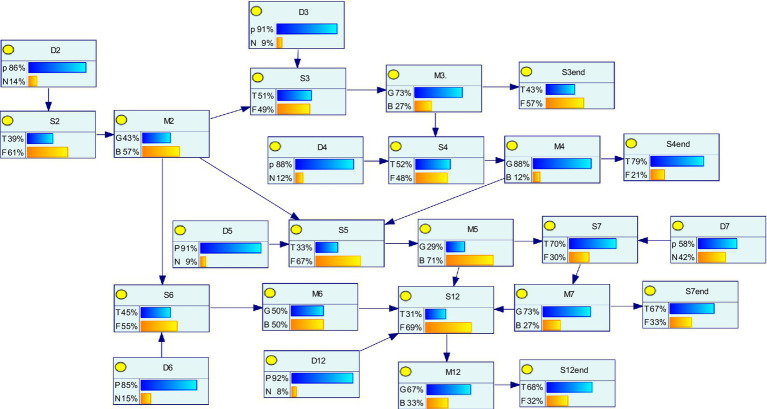
Network diagram of scenario evolution of major outbreak dynamics in 2020.

The state probability of each node variable was calculated by Equations 1–4, via the Bayesian joint probability formula, which was obtained via GeNIe software, as shown in Figure 9.

Figure 9 illustrates the scenario Bayesian network which is established by combining the prior probabilities of each response variable. Using the Bayesian joint probability formula, the state probabilities of the response variables in each scenario stage are calculated. Through the report cases, the range of values, node state probabilities and the scenario Bayesian network for each scenario are determined by combining expert scores based on the understanding of response measures and response results.

### 4.3 Sensitivity analysis

Bayesian network sensitivity analysis is achieved by studying the effects of small changes in the numerical parameters of the model (i.e., prior and conditional probabilities) on the output parameters (posterior probabilities). This approach is useful for identifying key parameters and dependencies. Highly sensitive parameters have a more significant impact than other parameters on the model push results, and identifying them allows for the targeted allocation of work. Sensitivity analyses of Bayesian networks are critical in fields such as risk assessment, healthcare, and reliability engineering.

**Figure 10:**
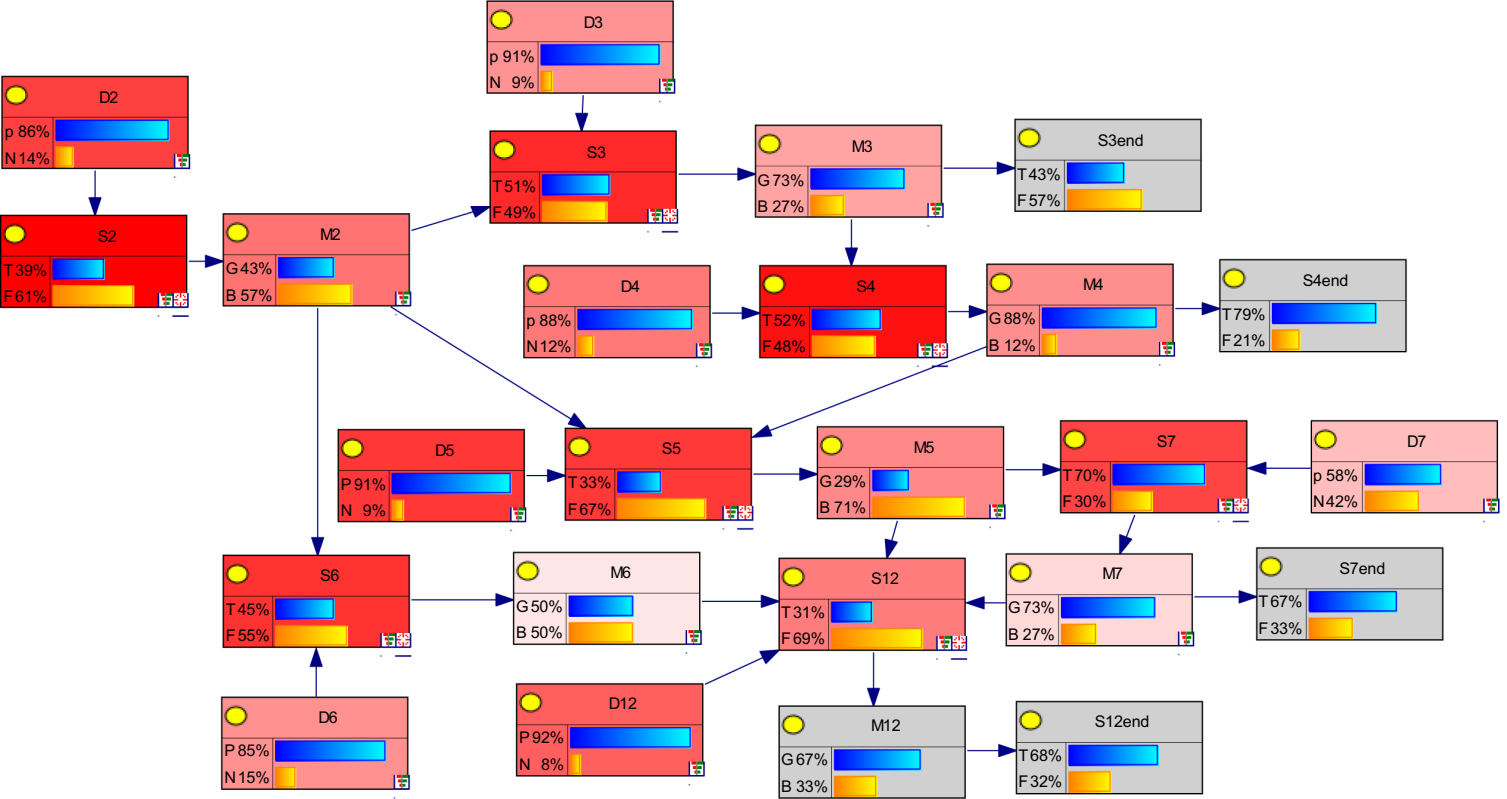
Schematic diagram of the Bayesian network sensitivity analysis for NCPHEs.

The nodes in the Bayesian network represent the individual scenarios that may occur in NCPHEs and various emergency response measures. To identify the emergency response measures that have a the greatest impact on each scenario, the scenario nodes S2, S3, S4, S5, S6, S7, and S12 are defined as target nodes. The sensitivity analysis results for this group of nodes are shown in Figure 10.

In this Figure 10, darker-colored nodes indicate higher sensitivity. Notably, the emergency response measure nodes M2, M3, M4, and M5 exhibit particularly high sensitivity, meaning that small changes in these nodes can significantly influence the Bayesian-derived scenario network. Based on these findings, we recommend prioritizing these four measures in the emergency management of NCPHEs: (1) temporarily closing high-exposure sites, (2) improving medical supplies, (3) strengthening protective measures for medical personnel, and (4) dispatching medical personnel in a timely manner.

## 5 Discussion

Since evolution of major public health events is a complex dynamic process affected by multiple factors, each key scenario node along an evolution path is associated with different emergency measures and treatment goals; thus, different emergency measures taken by emergency decision makers may lead to different scenario evolution paths. In the actual evolution of public health events, owing to their uncertainty and dynamism, there are often two directions of event evolution, namely, expected and unexpected, and these two directions have states of optimism and pessimism, respectively. Therefore, to ensure that major public health events develop along the path of the optimal scenario and reduce the damage and disruptions caused by evolution toward secondary scenarios, active and effective emergency measures and treatment goals are essential.

During the process of a major public health event, i.e., from occurrence to spread, the probabilities associated with scenario states S2, S3, S4, S5, S6, S7, and S12 are 38.8, 51, 52.4, 33.5, 45, 69.9, and 30.7%, respectively. The fluctuations among scenarios are obvious, indicating that emergency management, as well as the treatment objectives, have an effect on each scenario over time. In summary, the dynamic scenario evolution network of major epidemics basically conforms to the actual crisis spreading process, thereby confirming the effectiveness and feasibility of the model.

## 6 Conclusion

1. In this study, typical categories of NCPHE scenarios are summarized. There is no consensus regarding the scenario elements of NCPHEs. Thus, 26 typical spreading scenarios and 41 derivative scenarios related to public health emergencies were extracted from historical case information. The applied approach overcomes the ambiguity of scenario descriptions via text for public health events and provides scientific and detailed scenario elements for analyses.
2. A scenario-based statistical analysis of NCPHEs was performed with the proposed Bayesian network method to obtain scenario evolution patterns. On the basis of the “scenario–response” model and the SDM model, a Bayesian network for major epidemics was constructed. This approach effectively considers the uncertainty in the scenario evolution process, with important theoretical and practical significance for emergency decision makers to control the development of public health events and implement emergency response measures in a timely and effective manner for key scenario nodes. This study also provides scientific support for emergency management by relevant departments.
3. Due to the large number of subjects involved and the complexity of influencing factors in the emergency response process of a major public health event, to determine the conditional probability for each scenario node, we adopted fuzzy set theory and improved D–S evidence theory to reduce the subjectivity of expert scores. To reduce the risks associated with public health event crises, an interdisciplinary, cross-sectoral, and multilevel strategy is needed to provide effective assistance. In the future, we can do this by creating a database of scenarios of major public health events, encompassing both historical incident scenarios and frequently occurring scenarios. Moreover, to improve the autonomous learning capability of the proposed model, the Bayesian network can be automatically updated when new scenario data are generated.

Although combining the scenario approach and the Bayesian network model allowed us to obtain useful predictions given changes in public health event dynamics, the most important elements of a complex system of public health outbreaks are the multiple and interacting drivers of the disease itself; these drivers are difficult to predict, such as the characteristics of the ever-mutating pathogens and the behavior of the human population in relation to resistance to the disease.

Therefore, two important topics will be explored in future studies. The first concerns the optimization of the Bayesian network. We plan to integrate real-time data streams from public health surveillance systems, social media, and other real-time data sources to compose new datasets. To improve the model’s adaptability, we propose incorporating temporal elements into the framework, enabling the model to track changes in scenario states over time. Dynamic Bayesian networks (DBNs) can be applied to assess temporal sequences of events and forecast how scenarios evolve in future time steps. We also propose embedding machine learning algorithms in the framework to enable DBNs to learn from new data and automatically adjust their probabilistic assumptions. The second optimization goal is to combine GIS systems with DBNs. Localized patterns of scenario evolution are simulated by adding spatially diverse environmental factors (climate, air quality, etc.) and demographic information to predict how interventions may have different impacts in specific regions.

## Data Availability

The original contributions presented in the study are included in the article/Supplementary material, further inquiries can be directed to the corresponding author.
