# Supplementary material for: Scenario construction and evolutionary analysis of nonconventional public health emergencies based on Bayesian networks
Source: Front Public Health. 2025 Feb 7;13:1489904. doi: 10.3389/fpubh.2025.1489904 (PMC11842347; doi:10.3389/fpubh.2025.1489904)
Supplement: Supplementary file 1 [file Table_1.doc]

**Appendix A**

Description of the scenarios underlying the spread of major public health events

| **Number** | **Scenario name** | **Scenario Description** |
| --- | --- | --- |
| S1 | Edible wild animals | Globally, over 300 zoonotic infectious and parasitic diseases have been identified, with animals being the source of approximately 60% of human infectious diseases. Additionally, 75% of these diseases are transmissible to humans. Increased human encroachment and exploitation further elevate the risk of such diseases. |
| S2 | Cluster epidemic: the local epidemic is severe and the number of infections increases sharply | NCPHEs in densely populated areas with inadequate health infrastructure, such as slums or tribal communities, frequently affect various settings including medical institutions, schools, and commercial spaces. However, the level of exposure within households is significantly higher compared to that of public gatherings or dining events. Inadequate and delayed isolation measures, coupled with an imbalanced response, can exacerbate these challenges, potentially triggering a "perfect storm" of public health crises in these regions. |
| S3 | Shortage of medical supplies and insufficient protective equipment | Medical resources are constrained, and managerial conflicts are prevalent, with both situations being exacerbated by a surge in infections that sharpens the mismatch between supply and demand. |
| S4 | Medical staff or police officers are sick or infected with the epidemic | Frontline medical and police personnel face heightened infection risks due to close interactions with the public and potentially infected individuals. Outbreaks within these groups can lead to staff shortages, strain healthcare and law enforcement systems, and amplify community transmission risks by diminishing the enforcement of health guidelines and emergency response capabilities. |
| S5 | Insufficient supply of medical personnel and police personnel | Insufficient medical and law enforcement staff can result in delayed emergency responses, treatment, increased crime, and poor public health regulation enforcement, exacerbating public safety and health issues and reducing public trust. |
| S6 | Insufficient hospital beds | This scenario may cause significant treatment delays for patients, forcing medical staff to prioritize treatment based on severity and prognosis upon hospital admission. Emergency services might be redirected to other hospitals, exacerbating regional medical resource shortages and potentially deteriorating overall health due to delayed or insufficient care. |
| S7 | Misdiagnosis of virus carriers | This scenario may result in continued transmission, increasing infection rates. Furthermore, misdiagnosis may lead to incorrect treatments, adversely affecting patient health. |
| S8 | Recovered patient's "secondary infection" | Reinfection in recovered patients may suggest viral mutation or short-lived immunity, raising doubts about treatment and vaccine efficacy and challenging the concept of herd immunity. |
| S9 | Asymptomatic virus carriers | Asymptomatic carriers can transmit the virus unnoticed, complicating the identification and isolation of carriers and heightening the risk of extensive spread, particularly in dense settings. Research using mobility data, networked dynamic metapopulation models, and Bayesian inference indicates that 79% of infections originate from asymptomatic individuals. |
| S10 | Expansion of virus transmission routes | When viral transmission pathways expand, it means the virus is able to spread over a wider range, potentially leading to a faster and wider spread of the disease. |
| S11 | Virus carriers conceal their illness and travel history, and citizens conceal their itinerary | Due to the high costs of treatment and isolation, and fear of impacting work and social interactions, some virus carriers may conceal their symptoms, travel history, or exposure. This can hinder timely tracking of their close contacts, complicating epidemiological investigations, exacerbating the epidemic's spread, and increasing community transmission risks. |
| S12 | Superspreading events caused by virus carriers | In epidemiology, a superspreader is defined as an individual who transmits an infectious disease to many others with exceptional efficiency. |
| S13 | Virus carriers without awareness of prevention and control | Asymptomatic individuals may not realize their infection status due to personal unawareness, limited epidemic knowledge, or scarce medical resources, preventing timely preventative actions or medical consultation. |
| S14 | Food (cold chain transportation) poses a risk of viral infection | In food production, packaging, or transportation, substandard hygiene, cross-contamination, exposure to contaminants, or virus-carrying workers can lead to food contamination. |
| S15 | Cases of cross-epidemics of other viruses such as influenza and epidemics at the same time | Individuals coinfected with influenza and another similar-symptom virus, such as coronavirus, can increase transmission risks and complicate epidemic management due to dual infection. |
| S16 | Virus mutation | Over time, as infections increase, viral genomes may mutate during replication across individuals, resulting in mutations, recombinations, or deletions. These genetic changes can alter the virus's characteristics, including transmission rate, infectivity, and clinical symptoms. |
| S17 | Lack of public health knowledge | Public knowledge gaps in daily public health prevention, such as proper mask usage and disinfection techniques, the understanding of emerging diseases such as the new coronavirus, vaccine effects and safety, and access to accurate health information and medical services contribute to inadequate public health responses. |
| S18 | Inappropriate remarks and rumors mislead the public | In the self-media era, rumors in public health emergencies exhibit traits such as diverse dissemination channels, fragmented content, real-time speed, complex motives, and global scope. Creating and deliberately spreading misinformation, along with inappropriate comments, can amount to hate speech, insults, defamation, or incitement, thereby negatively impacting social and public health security. |
| S19 | Manufacture and sale of counterfeit goods, manufacture and sale of counterfeit related medical supplies | During the epidemic, increased demand for medical supplies led to the production and sale of counterfeit products, including low-quality masks, ineffective disinfectants, and fake vaccines, as well as substandard drugs and medical equipment. Such behavior not only directly jeopardizes public health but also erodes trust in medical products and systems, and disrupts the formal market. |
| S20 | Insufficient vaccine supply | Vaccine supply challenges, including insufficient production capacity, raw material shortages, unexpected high demand, and distribution problems, are related to issues in mass production, storage, and the entire upstream and downstream supply chain, such as production of vaccine vials, rubber stoppers, and labels, as well as cold chain logistics, temperature control, dry ice requirements, and air cargo difficulties. |
| S21 | privacy leakage issue | During epidemic control, governments and public health bodies rapidly gather vast data, including sensitive personal health, location, and contact information. Security flaws in data collection apps may expose user data, risking incorrect disclosure of personal health details, potentially leading to social discrimination or career disadvantages. |
| S22 | Disposal of infected animals and animals involved in epidemics | Infected animals refer to those animals that have been confirmed to be infected with a certain pathogen, while associated animals may include those potentially infected or at risk due to connections with known epidemics, including pet disposal. The World Organization for Animal Health reports that since the outbreak, over 20 countries including the US, UK, Argentina, Brazil, Canada, and France have documented cases of cats and dogs with the new coronavirus. |
| S23 | Mental health issues of virus carriers | Upon diagnosis, individuals with the virus may initially feel shock and unease, driven by fear of the unknown, health concerns, and guilt about potentially endangering others. As the illness progresses, the stress of necessary social and familial distancing can intensify feelings of isolation. Additionally, the virus may directly impact neurological functions, including the central nervous system. |
| S24 | Natural disasters during epidemic times | Natural disasters such as earthquakes, floods, hurricanes, or fires can undermine disease control efforts by damaging infrastructure, disrupting utilities, and destroying homes and medical facilities. These events necessitate extensive emergency responses, straining resources already limited by epidemics, such as medical supplies and manpower, potentially reducing disaster relief effectiveness and efficiency. Additionally, rescue workers and volunteers face increased health risks during these operations |
| S25 | patients return to positive after recovery | The phenomenon of retesting positive may result from incomplete viral clearance or a weakened immune response. Such cases challenge public health efforts as these individuals may unknowingly contribute to viral transmission, posing a risk of further epidemic spread. |
| S26 | Overseas imported cases | Individuals testing positive after international travel can become potential sources of epidemic spread. Imported cases, such as those resulting from crossing borders during the incubation period, risk causing multicountry outbreaks. Without timely intervention, the number of new overseas cases may surpass the number of local incidents. |

| **Scenario number** | **Scenario name** | **Source event scenarios** |
| --- | --- | --- |
| A1 | Inadequate implementation of preventive and control measures or improper performance of duties by relevant entities | S2, S4, S5 |
| A2 | Significant impact on certain businesses or industries | S2 |
| A3 | Impact on the livelihood security of uninfected people | S2 |
| A4 | Excessive wear and tear on prevention and control measures | S2, S3 |
| A5 | Mask contamination | S2, S3, S17, S19 |
| A6 | Negative emotions from the epidemic | S2, S11, S21, A7 |
| A7 | Social security problems caused by the epidemic | S3, S18, S19 |
| A8 | Management of excess medical waste during epidemics | S3 |
| A9 | Noncompliance or obstruction of population to prevention and control measures | S4, S5, S11, S17, S18 |
| A10 | Donation issues | A1 |
| A11 | Employment discrimination against recovered and asymptomatic individuals. | S17, S18, S21 |
| A12 | Vaccination hesitation | S18, S19 |
| A13 | Management malpractice during the epidemic | S4 |
| A14 | Inadequate prevention and control of special populations such as nursing homes and kindergartens | S3, S4 |
| A15 | consumer protection | S19 |
| A16 | Transport of infected bodies | S2, S3 |
| A17 | The problem of incinerators for the disposal of dead bodies | S2, S3 |
| A18 | Products, enterprises seize epidemic-related trademarks | S3, S19 |
| A19 | bid up prices | S3, S19 |
| A20 | Rental issues | S2 |
| A21 | Sterilizing drug abuse | S17, S18 |
| A22 | Impact on the operation and development of healthcare institutions | S2, S23 |
| A23 | Unemployment as a result of the epidemic, affecting jobs | S17, S18, A2 |
| A24 | Agriculture and marketing of agricultural products affected | S2, S12 |
| A25 | Overprotection against epidemics | S11, A1, |
| A26 | Problems of school education | S2 |
| A27 | Medication for chronic diseases or other patients | S3, S4 |
| A28 | Volunteer honor issues | S3, S4, S5, S18 |
| A29 | Discriminations against persons in infected areas | S17, S18, S21 |
| A30 | Overinspection of prevention and control services. | S4, S11 |
| A31 | Inconsistent data reporting standards and redundant tabulation | S4, S5, A1 |
| A32 | Insufficient supply in the upstream and downstream of the protective materials industry chain | A2 |
| A33 | Foreign trade affected | A2 |
| A34 | The public is overloaded with information that cannot be filtered | A1, S17, S18 |
| A35 | Public hoarding | S17, S18, S23 |
| A36 | Stress or rejection of emergency facilities by residents | S3, S6 |
| A37 | The Negative Impact of Online Education on Educators | S2, A14, A26 |
| B1 | Promoting online consumption | S2, A24 |
| B2 | Promoting the development of new online businesses | A2 |
| B3 | Online/telecommuting models optimized | A1, A3, |
| B4 | Decrease in the number of criminal cases | S2, S21, A30 |

**Appendix B**

Mathematical exposition

Assuming that x is the set of parent nodes or the set of causal relationships in a BN and that y is the result (called the child node in a BN causal relationship structure), we have $x\to y$, where the set x contains n elements, each of which is denoted as $x_{i}$. Then, we have $x_{i}\in x(i=1\text{，}2\text{，…，n)}$, and the set y contains n elements, each of which is denoted as $y_{i}$. The full probability formula yields the probability of the occurrence of an outcome if the cause is known, as follows:

$P(y)=P(xy)=P(x_{1}y+x_{2}y+\text{…}+x_{n}y)=P(x_{1}y)+P(x_{2}y)+\text{…}+P(x_{n}y)$ (1)

From the full probability formula, it is evident that the posterior probability of a child node can be determined via the prior probability of the parent node and the conditional probability from the parent to the child node. Conversely, the Bayesian formula provides a means to infer the probability of a cause on the basis of a known outcome. This approach essentially reverses the full probability formula and is expressed in the following form:

$P(x_{i}\left|y\right.)=\frac{P(x,y)}{P(y)}=\frac{P(x_{i})P(y\left|x_{i})\right.}{\sum_{j=1}^{n} P(x_{j})P(y\left|x_{j})\right.}$ (2)

In a BN model, if the set of parent nodes of a particular node is established and given the conditional independence assumption inherent to BNs, then the node in question is statistically independent of all nondescendant nodes. Under these conditions, the joint probability distribution can be formulated as follows:

$P(x_{1},x_{2},\text{…,}x_{n})=\prod_{i=1}^{n} P(x_{1}\left|P(\prod x_{n}\right.))$ (3)

To overcome the limitations of expert knowledge and the influence of personal preferences on the accuracy of empirical probability, Dempster–Shafer (DS) theory[[[1]](#endnote-2)] is used to integrate the empirical probability values ​​and uncertain information given by experts to reduce their subjective effects and ensure the reliability and rationality of node probabilities.

Dempster's law of synthesis requires the integration of multiple independent pieces of evidence m_1_, m_2_,... m_n_, which is the core of D–S evidence theory[[[2]](#endnote-3)]. It is assumed that there are n experts evaluating the indicator system as follows:

$M\text{=}\left(\begin{matrix} M_{1} \\ M_{2} \\ M_{3} \\ \vdots\\ M_{n} \end{matrix}\right)=\left(\begin{matrix} m_{11} & m_{12} & \cdots& m_{15} \\ m_{21} & m_{22} & \cdots& m_{25} \\ m_{31} & m_{32} & \cdots& m_{35} \\ \vdots& \vdots& & \vdots\\ m_{n1} & m_{n2} & \cdots& m_{n5} \end{matrix}\right)$ (4)

m_ij_ in Formula (4) represents the basic probability assignment of the i-th expert to the j-th evaluation indicator. The sum of all these probabilities in M ​​is 1.

Matrix R is obtained via a product operation, namely, the transpose of the i-th row MjT is multiplied by the j-th row Mj:

$A=M_{i}^{T}\times M_{j}\text{=}\left(\begin{matrix} m_{i1} \\ m_{i2} \\ m_{i3} \\ m_{i4} \\ m_{i5} \end{matrix}\right)\left(m_{j1}m_{j2}m_{j3}m_{j4}m_{j5}\right)=\left(\begin{matrix} m_{i1}\times m_{j1} & \cdots& m_{i1}\times m_{j5} \\ \vdots& \ddots& \vdots\\ m_{i5}\times m_{j1} & \cdots& m_{i5}\times m_{j5} \end{matrix}\right)$ (5)

The sum of the probabilities of the main diagonal elements in Matrix A is the numerator in Dempster’s composition equation shown in Formula (6), and the sum of the probabilities of the nonmain diagonal elements of all of the matrices obtained in each fusion process is the conflict degree K after fusion, that is, the degree of conflict among the n pieces of evidence.

Finally, an improved version of the D–S evidence theory synthesis algorithm considering the weight distribution is used to calculate the fused probability value. The improved fusion formula is as follows [[[3]](#endnote-4)]:

$\begin{matrix} m_{DS}(A)=\left\{\begin{matrix} \frac{\sum_{A_{i}\cap B_{j}\cdots=A} m_{1}(A_{i})m_{2}(B_{i})}{1-K}+\text{f(A) }A\neq\emptyset\\ \text{ }0\text{ }A=\emptyset\end{matrix}\right. \\ \end{matrix}$ (6)

f(A)=k•q(A) in the formula is the probability distribution function of evidence conflict; that is, the conflict degree K between each piece of evidence is distributed to each element in Matrix A. Therefore, the probability distribution formula satisfies $\sum_{A\subset\theta}f\left(A\right)\text{=}K$, where $q\left(A\right)=\frac{\sum_{i=1}^{n}m_{i}(A)}{n}$allocates K to A according to this ratio.

The conditional probability of each situation state node Si is obtained and substituted into Formula (3). P_a_(S_i_) represents the parent node set of the situation state node Si, and the corresponding formula can be expressed as follows:

$P(S_{1},S_{2},\cdots,S_{n})=\prod_{i=1}^{n} P(S_{i}\left|P_{a}(S_{i}\right.))$ (7)

**Appendix C**

Calculation of probability of all nodes.

| **Probability Calculation** | **Prior Probability** | **Conditional Probability** |
| --- | --- | --- |
| P（S2） | P(D2=P)=0.863  P(D2=N)=0.137 | P(S2=T∣D2=P)=0.32968  P(S2=T∣D2=N)=0.75815 |
| P（S3） | P(D3=P)=0.910  P(D3=N)=0.090  P(M2=G)=0.432  P(M2=B)=0.568 | P(S3=T∣D3=P,M2=G)=0.23898  P(S3=T∣D3=P,M2=B)=0.68356  P(S3=T∣D3=N,M2=G)=0.53264  P(S3=T∣D3=N,M2=B)=0.82792 |
| P（S4） | P(M3=G)=0.733  P(M3=B)=0.267  P(D4=P)=0.880  P(D4=N)=0.120 | P(S4=T∣M3=G,D4=P)=0.3919  P(S4=T∣M3=G,D4=N)=0.7477  P(S4=T∣M3=B,D4=P)=0.7671  P(S4=T∣M3=B,D4=N)=0.7921 |
| P（S5） | P(D5=P)=0.909  P(D5=N)=0.091  P(M4=G)=0.884  P(M4=B)=0.116  P(M2=G)=0.432  P(M2=B)=0.568 | P(S5=T∣D5=P,M4=G,M2=G)=0.22302  P(S5=T∣D5=P,M4=G,M2=B)=0.25368  P(S5=T∣D5=P,M4=B,M2=G)=0.69165  P(S5=T∣D5=N,M4=G,M2=G)=0.71127  P(S5=T∣D5=N,M4=G,M2=B)=0.78365  P(S5=T∣D5=N,M4=B,M2=G)=0.6792  P(S5=T∣D5=N,M4=B,M2=B)=0.79568 |
| P（S6） | P(D6=P)=0.847  P(D6=N)=0.153  P(M2=G)=0.432  P(M2=B)=0.568 | P(S6=T∣D6=P,M2=G)=0.11614  P(S6=T∣D6=P,M2=B)=0.64820  P(S6=T∣D6=N,M2=G)=0.58327  P(S6=T∣D6=N,M2=B)=0.66260 |
| P（S7） | P(M5=G)=0.287  P(M5=B)=0.713  P(D7=P)=0.582  P(D7=N)=0.418 | P(S7=T∣M5=G,D7=P)=0.29422  P(S7=T∣M5=G,D7=N)=0.67784  P(S7=T∣M5=B,D7=P)=0.79272  P(S7=T∣M5=B,D7=N)=0.80402 |
| P（S12） | P(D12=P)=0.923,P(D12=N)=0.077  P(M5=G)=0.288,P(M5=B)=0.712  P(M6=G)=0.498,P(M6=B)=0.502  P(M7=G)=0.728,P(M7=B)=0.272 | P(S12=T∣D12=P,M5=G,M6=G,M7=G)=0.20717  P(S12=T∣D12=P,M5=G,M6=G,M7=B)=0.23134  P(S12=T∣D12=P,M5=G,M6=B,M7=G)=0.27320  P(S12=T∣D12=P,M5=G,M6=B,M7=B)=0.35107  P(S12=T∣D12=P,M5=B,M6=G,M7=G)=0.25739  P(S12=T∣D12=P,M5=B,M6=G,M7=B)=0.30993  P(S12=T∣D12=P,M5=B,M6=B,M7=G)=0.26957  P(S12=T∣D12=P,M5=B,M6=B,M7=B)=0.45409  P(S12=T∣D12=N,M5=G,M6=G,M7=G)=0.78650  P(S12=T∣D12=N,M5=G,M6=G,M7=B)=0.70280  P(S12=T∣D12=N,M5=G,M6=B,M7=G)=0.79019  P(S12=T∣D12=N,M5=G,M6=B,M7=B)=0.71991  P(S12=T∣D12=N,M5=B,M6=G,M7=G)=0.31662  P(S12=T∣D12=N,M5=B,M6=G,M7=B)=0.24521  P(S12=T∣D12=N,M5=B,M6=B,M7=G)=0.79918  P(S12=T∣D12=N,M5=B,M6=B,M7=B)=0.86475 |

1. [?]Yaghubi Agreh O , Ghaffari-Hadigheh A . Application of Dempster-Shafer theory in combining the experts' opinions in DEA[J]. Journal of the Operational Research Society, 2018:1-1 [↑](#endnote-ref-2)
2. [?] Sentz K, Ferson S (2002) Combination of evidence in Dempster–Shafer theory, vol 4015. Sandia National Laboratories, Albuquerque. https://doi.org/10.2172/800792 [↑](#endnote-ref-3)
3. [?] Yager R (1987) On the Dempster–Shafer framework and new combination rules. Inf Sci 41(2):93–137. https://doi.org/10.1016/0020-0255(87)90007-7 [↑](#endnote-ref-4)
